# Identification of Antifungal Compounds Active against *Candida albicans* Using an Improved High-Throughput *Caenorhabditis elegans* Assay

**DOI:** 10.1371/journal.pone.0007025

**Published:** 2009-09-14

**Authors:** Ikechukwu Okoli, Jeffrey J. Coleman, Emmanouil Tempakakis, W. Frank An, Edward Holson, Florence Wagner, Annie L. Conery, Jonah Larkins-Ford, Gang Wu, Andy Stern, Frederick M. Ausubel, Eleftherios Mylonakis

**Affiliations:** 1 Division of Infectious Diseases, Massachusetts General Hospital, Boston, Massachusetts, United States of America; 2 Broad Institute of Massachusetts Institute of Technology and Harvard, Cambridge, Massachusetts, United States of America; 3 Department of Molecular Biology, Massachusetts General Hospital, Boston, Massachusetts, United States of America; University of Missouri-Kansas City, United States of America

## Abstract

*Candida albicans*, the most common human pathogenic fungus, can establish a persistent lethal infection in the intestine of the microscopic nematode *Caenorhabditis elegans*. The *C. elegans*–*C. albicans* infection model was previously adapted to screen for antifungal compounds. Modifications to this screen have been made to facilitate a high-throughput assay including co-inoculation of nematodes with *C. albicans* and instrumentation allowing precise dispensing of worms into assay wells, eliminating two labor-intensive steps. This high-throughput method was utilized to screen a library of 3,228 compounds represented by 1,948 bioactive compounds and 1,280 small molecules derived via diversity-oriented synthesis. Nineteen compounds were identified that conferred an increase in *C. elegans* survival, including most known antifungal compounds within the chemical library. In addition to seven clinically used antifungal compounds, twelve compounds were identified which are not primarily used as antifungal agents, including three immunosuppressive drugs. This assay also allowed the assessment of the relative minimal inhibitory concentration, the effective concentration *in vivo*, and the toxicity of the compound in a single assay.

## Introduction

The soil dwelling nematode *Caenorhabditis elegans* has become a model host to study mammalian virulence of both bacterial and fungal pathogens [1], [2], including *Candida albicans*, the most commonly isolated fungal pathogen from blood cultures [3]. Importantly, key components of *Candida* pathogenesis in mammals, such as biofilm and filament formation, are also involved in nematode killing [2]. Because of these features, assays have been devised that involve killing of *C. elegans* by fungal pathogens which can be used to screen fungal mutants for virulence factors and libraries of compounds for antifungal activity [4]–[6].

Use of a whole animal assay during antifungal screens provides several advantages when compared to more commonly used inhibitory compound screens that are preformed *in vitro*. First, the immunomodulatory affect of the compound on the host immune system can be assessed in the presence of a pathogen. Additionally, effective compounds that function by inhibiting pathogen virulence factors can be identified, as some of these factors are only expressed in a host. Another important advantage of using a model host during screening is that it allows simultaneous assessment of toxicity. Frequently, compounds toxic to fungi also have undesirable effects on the mammalian host. This toxicity/efficacy “bottleneck” delays antifungal drug discovery.

*C. elegans* whole-animal screens in particular have several advantages over other mammalian screening models. The *C. elegans* model does not raise as many ethical concerns as the use of vertebrate models. Moreover, nematodes are easy to manipulate in the laboratory because they are small enough to fit into standard 96- and 384-well microtiter plates and they have short and simple reproductive cycles. *C. elegans* worms are also relatively inexpensive to propagate in large numbers and are genetically tractable [7]–[9]. The two main objectives of this paper are to first develop an automated, high-throughput, *C. elegans* assay that can be used to screen chemical compounds *in vivo*, and second use this new assay to identify chemicals with antifungal activity.

## Methods

### Media and strains

*C. albicans* strain DAY185 [10], which was used in all assays, was grown overnight to late-log phase with shaking at 30°C in yeast extract peptone dextrose (YPD) (Difco) medium containing the antibiotics kanamycin (45 µg/ml), ampicillin (100 µg/ml), and streptomycin (100 µg/ml). The cells were centrifuged, washed with PBS and re-suspended at a concentration of 2.5×10^5^ cells/ml.

A *C. elegans glp-4;sek-1* double mutant was used in all assays. The *glp-4(bn2)* mutation renders the strain incapable of producing progeny at 25°C [11] and the *sek-1(km4)* mutation causes enhanced sensitivity to various pathogens [12], thereby decreasing assay time. Nematodes were grown on nematode growth medium (NGM) with *Escherichia coli* strain HB101 as the food source as previously described [6], [13]. Screen medium was 30% brain heart infusion medium (BHI, Difco) in M9 buffer [13] containing antibiotics kanamycin (90 µg/ml), ampicillin (200 µg/ml), and streptomycin (200 µg/ml). M9 buffer was used to wash the worms as needed and for diluting screen media.

### Z′-factor values

Positive and negative control data were used to calculate the Z′-factor as a measure of overall assay quality [14]. Z′ = 1−((3σ_c+_+3σ_c−_)/(|μ_c+_−μ_c−_|)), where μ_c+_ and μ_c−_ are the averages of test sample signals for both the positive and negative controls respectively, and σ_c+_ and σ_c−_ are the variations in signal measurements for the positive and negative controls respectively. Dimethyl sulfoxide (DMSO, 2%) in the screen medium served as the negative control, and amphotericin B and ketoconazole in screen medium were used individually as positive antifungal controls. Concentrations of the antifungal agents used were effective against the *C. albicans* DAY185 strain used for the assay. Two columns (16 wells) in each 96-well plates were used for each concentration of the antifungal. Z′-factor values were calculated for each of the antifungal agents.

### *C. elegans–C. albicans* assay

The pre-infection assay was performed as previously described [6]. In brief, worms grown on NGM plates were washed with M9 buffer and placed on 48 h-old *C. albicans* lawns (on BHI agar plates) for 2 h. The worms were washed off the plates using screen medium, transferred to two separate beakers, and re-suspended at a density of 1–2 worm/µl in screen medium. Twenty µl of the suspension of pre-infected worms were added to wells of 96-well non-binding half area plates (Corning) containing 80 µl of screen medium. The details concerning the addition of compounds to the wells are stated below in the following text. Plates were sealed with Breathe-Easy™ membranes (Diversified Biotech), and incubated at 25°C for 96 h. The survival rates of both exposed and unexposed worms were analyzed using the STATA 6 software.

The procedures for the co-inoculation assay were similar to those of the pre-infection assay above with the following modifications [15]: 70 µl of screen medium was dispensed into each well of a 96-well non-binding half area plate (Corning) using a MultiDrop Combi Plate Filler (Thermo Scientific) equipped with a standard dispensing cassette (Thermo Scientific). When compounds were used, approximately 450 nl of each compound was pin-transferred from compound plates into individual wells (containing 70 µl of screen medium) of 96-well assay plates simultaneously with a 96-pin head (CyBi^®^-Well, CyBio). Worms were grown and washed as in the pre-infection assay, except that they were not previously infected with *C. albicans*. A Union Biometrica COPAS-BIOSORT^TM^ worm sorter was used to dispense automatically 15+/−1 worms into each well, followed by addition of 10 µl of a *C. albicans* suspension at a density of 2.5×10^5^ cells/ml in PBS with a Multidrop Combi Plate Filler.

### Acquisition and analysis of data for co-inoculation assay

At the end the incubation period, the plates were imaged using the Molecular Devices Discovery-1 microscope (MDS Analytical Technologies) running MetaExpress software by capturing transmitted light images. Images of the entire well were captured using a 2× low magnification objective lens. Prior to analysis, the images were converted from 16 bit TIF files to 8 bit JPEG files using the CellProfiler™ image analysis software [16], [17]. The resulting images were visually analyzed for *in vitro* fungal growth, followed by visually scoring of live and dead worms based on nematode shape, as live worms appear sinusoidal and dead worms are rod shaped.

The determination of the lowest concentration of the selected compounds showing *in vitro* antifungal activity was accomplished by following the steps detailed in the co-inoculation assay, using two-fold serial dilutions of the test compounds. The wells were assessed by visually monitoring the turbidity for concentrations exhibiting *in vitro* inhibition of *C. albicans* growth. A quantitative scoring system was developed to measure and rank the activity of hits. For this, compounds of interest were tested in a series of two-fold dilutions, and the half maximum effective concentrations (EC_50_) which conferred 50% survival of the worms was determined.

## Results/Discussion

### Optimization of the *C. albicans*–*C. elegans* assay for high-throughput use

Previous work has demonstrated that *C. elegans* can serve as a host for *Candida* spp. and an infection is established in the worm intestine when nematodes are fed on a lawn of *C. albicans* on agar medium [6]. Using this pathosystem, a *C. elegans-C. albicans* “curing” assay was developed using liquid media in standard 96-well plates which allowed simultaneous assessment of both the compound's antifungal activity and the compound's cellular toxicity. This relatively low-throughput assay entailed pre-infection of the nematodes, by placing them on a *C. albicans* lawn on BHI agar plates prior to the assay, followed by manually pipetting the infected worms into microtiter plates containing the compounds to be screened.

**Figure 1:**
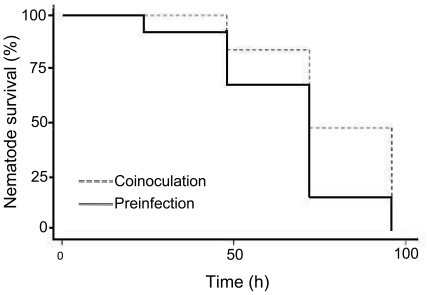
Comparison of inoculation procedures. Comparison of nematode survival between worms co-inoculated with *C. albicans* during compound exposure, and worms pre-infected with the fungus prior to compound exposure.

One of the goals of the current work was to increase the throughput of the chemical screening assay by utilizing a commercial instrument to distribute a precise number of infected worms in each microtiter well. However, the use of the instrument to dispense *Candida*-infected nematodes was not practical, as the fungus formed avid biofilms on the surface of the instrument such that it could not be efficiently decontaminated after each use. To circumvent this problem, direct *C. albicans* infection of the worms by co-inoculation in assay wells containing the liquid medium was assessed. The survival of worms pre-exposed to *C. albicans* lawns on agar before transferring to the assay medium was compared to worm survival when the nematode and *C. albicans* were co-inoculated in the assay medium. The rate of worm survival was comparable in the co-inoculation and pre-infection assays (Figure 1), demonstrating that prior exposure of the nematodes to *C. albicans* on solid media is not necessary for killing of the nematodes. The killing of worms may commence sooner when worms are pre-infected for two hours before transferring to assay wells, but by four days there was no significant difference between the two treatments, as all of the worms were dead in both instances. Additionally, there was no difference in the frequency and amount of filamention by *C. albicans* in dead worms in either assay (data not shown).

Several concentrations of fungal inoculum (10^2^ to 10^5^ cells/well) were examined, and a concentration 2.5×10^3^ cells/well was optimal for the 96 hour assay period, which resulted in a killing rate comparable to the pre-infection assay (data not shown). Higher concentrations were equally effective in killing, but resulted in wells with high turbidity that required a washing step prior to imaging in order to score the assay results. Fifteen worms per well of the 96-well half area plate was optimal for the assay, as wells with more than 15 worms per well caused the worms to overlap in the confined area making it more difficult to score whether the nematodes were alive or dead based on shape.

In the *C. elegans*-*C. albicans* antifungal discovery assay detailed in Breger et al (2007) [6], preinfection of the worms requires not only additional time to allow ingestion and subsequent colonization of the nematode intestinal tract, but also additional resources such as premade plates with lawns of *C. albicans* for the infection and materials to recollect the worms post-inoculation. This improved assay also cuts time by dispensing a precise number of worms by instrumentation instead of manual pipetting. As worms are manually pipetted they begin to settle at the bottom of the tube where they are held, creating a “gradient” of various worm densities where the top portion of the liquid contains a lower density of worms when compared to the bottom of the tube. This gradient leads to various amounts for worms being pipetted into each well, and thus creates situations where the assay for a compound may need to be repeated due to either too few or too many nematodes within each well. Therefore this improved assay not only saves several hours when setting up the assay, but may save several weeks as some compounds would not have to be repeated by additional screening.

### Evaluation of the co-inoculation assay

In order to evaluate the reproducibility and robustness of the co-infection assay the Z′-factor was determined. This calculation takes into account signals from both positive and background samples and is used to assess the probability that a sample scored as positive in a screen is genuine and not due to background noise [14]. For this evaluation, the percentage of worm survival was used in calculations after the following treatments: dimethyl sulfoxide (DMSO) was used as a negative control (background), and amphotericin B (2.5 µg/ml) and ketoconazole (16.0 µg/ml), (which conferred 93–100% worm survival) were used as the positive controls. In three independent experiments during assay development, the Z′ factor values were reproducible for each of the compound concentrations, ranging from 0.69 to 0.72, with an average value of 0.71.

**Figure 2:**
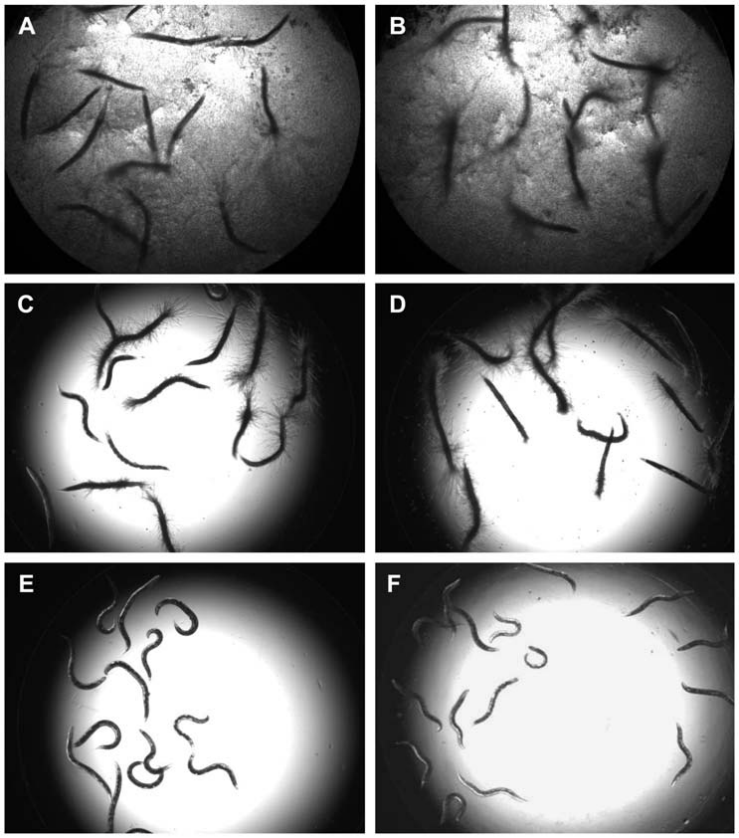
Representative assay wells from the *C. elegans*-*C. albicans* antifungal compound screen. (A) Negative control (DMSO added); (C) and (E) Amphothericin B was added as a positive control at either 1.0 µg/mL (C) or 2.5 µg/mL (E); (B,D,F) Screened compound (in this case ketoconazole) exhibiting no antifungal activity (2 µg/mL; (B)), *in vitro* antifungal activity (4 µg/mL; (D)), and *in vivo* antifungal activity conferring *C. elegans* survival (8 µg/mL (F).

Similar to the pre-infection assay, wells that contained no antifungal, compounds without antifungal efficacy, or antifungal compounds at a concentration below the effective dose result in rod-shaped dead worms with fungal filaments protruding from their cuticles (Figures 2A–2D). Interestingly, growth of *C. albicans* within the liquid medium was not necessary for nematode death, as filaments of *C. albicans* were seen extruding from some dead nematodes despite no *in vitro* growth, suggesting the compound inhibited the fungus *in vitro* but not *in vivo*. This observation was made in control assay wells, as treatment with 1 µg/ml of amphotericin B exhibited antifungal activity *in vitro*, but not *in vivo* (Figure 2C) as well as compounds identified in the screen (Figure 2D). However, in wells with effective antifungal agents at inhibitory concentrations, the worms maintain their sinusoidal shape, and the media remained free of fungal growth. This observation was made in both control assay wells with amphotericin B and well containing screened antifungal compounds exhibiting both *in vitro* and *in vivo* activities (Figure 2E and 2F).

During the preliminary screen of the compound library, wells that displayed a reduction or no *in vitro* fungal growth, however had little or no nematode survival, were counted and potential antifungal compounds. Upon confirmatory experiments, the compounds were able to confer nematode survival at higher concentrations than initially tested in the preliminary screening process. Therefore, all identified compounds conferred *in vitro* and *in vivo* antifungal activity. There were no compounds identified in the screen which conferred *C. elegans* survival in the presence of *in vitro* fungal growth, an observation which would suggest that the compound may have 1) an immunomodulatory effect on the nematode immune response, and/or 2) properties which inhibit pathogen specific virulence factor(s). Despite not finding any compounds which inhibit *C. albicans in vivo* but not *in vitro*, we feel it is possible to do so using this assay as the concentration of fungal cells initially inoculated to the wells still enables the visualization of the nematodes at the conclusion of the screen.

Of note is that in previous studies the scoring of live/dead and worms with and without filaments was accomplished by direct microscopic observation of movement and morphology of the nematodes. In these assays, digital images from the Discovery-1 microscope were captured and visually inspected for the analysis of assay results. This method improved the accuracy of analysis and evaluation of the assay, and moved the process closer to an automated “screening by imaging” system. Recently, SYTOX Orange live/dead staining has been utilized to facilitate high-throughput scoring of *C. elegans* survival [18], although the same approach may not necessarily be feasible for *C. albicans* due to background resulting from staining of live hyphal filaments extending from the dead nematode. Alternatively, imaging programs that can differentiate between nematode shapes can also possibly be developed to facilitate scoring of nematode survival at the conclusion of the screen [16], [17], [19].

### Identification of antifungal compounds resulting from the screen

**Figure 3:**
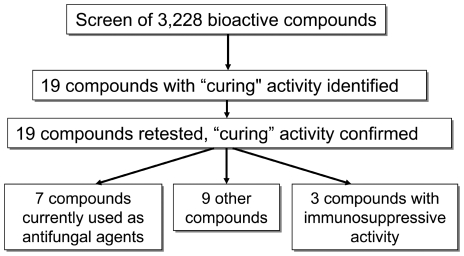
Flow chart representing the *C. elegans* “curing” assay that identified 19 effective compounds.

After establishing the robustness and repeatability of the assay, a screen of 3,228 compounds was conducted representing compounds not screened in previous studies. This study, in combination with the previous screen by Breger et al (2007), brings the total number of screened compounds to 4,494. This collection was represented by 1,948 bioactive compounds consisting of FDA approved drugs and other compounds from the Microscorce Spectrum collection (Microsource Discovery Systems, Inc.) and the Prestwick compound library (Prestwick Chemicals, Inc.) and 1,280 small molecules derived via diversity-oriented synthesis [20], [21] from the Broad Institute of Harvard and MIT (Cambridge, MA). As shown in Table 1, the screening library represented a diverse collection of bioactive compounds composed of multiple chemical classes containing a broad array of functionality. In total, 19 compounds were identified in the screen (Figure 3). More importantly, the *C. elegans* screen was able to detect most of the well established antifungal agents (Table 1 and Table 2).

**Table 1:** Compounds exhibiting antifungal activity identified in the *C. elegans*–*C. albicans* infection assay.

Compound	Action/Use	Reference(s)	
**Currently used antifungal agents**			
Amphotericin B	Interacts with ergosterol to form transmembrane channels	[47], [48]	
Nystatin	Binds to ergosterol to produce permeability changes	[49]	
Ketoconazole	Interferes with synthesis of ergosterol	[50]	
Terconazole	Interferes with synthesis of ergosterol	[51]	
Bifonazole	Interferes with synthesis of ergosterol	[50]	
Butoconazole nitrate	Interferes with synthesis of ergosterol	[52], [53]	
Oxiconazole nitrate	Interferes with synthesis of ergosterol	[52], [54]	
**Immunosuppressant agents**			
Ascomycin	Ethyl analogue of FK-506; binds to immunophilins, inhibits production of Th1 and Th2 cytokines		[55]
FK-506 (Tacrolimus)	Forms a complex with FKBP-12 to inhibit calcineurin		[56]
Cyclosporin A	Forms a complex with cyclophilin to inhibit calcineurin		[57]
**Other compounds**			
Triadimefon	Affects gibberellin and sterol biosynthesis		[39]
Dequalinium chloride	Potent anti-tumor activity and protein kinase C alpha (PKC) inhibitor		[58]
Valinomycin	Apoptosis inducer by disrupting mitochondrial membrane potential; affects morphology of *C. albicans*		[59]–[61]
Phenylmercuric acetate	Used as fungicide, herbicide, slimicide and bacteriocide; metabolized to diphenylmercury		[62]
Malachite green carbinol base	Malachite green is used to treat parasites, fungal, and bacterial infections in fish and fish eggs; metabolized to the carbinol form and then to the toxic leucomalachite green form.		[42], [63]
Concanamycin A	Potent and specific inhibitor of vacuolar-ATPase		[40]
Ellipticine	Potent anti-tumor agent, acts by DNA intercalation and/or inhibition of topoisomerase II		[64]
Mepartricin	Used to treat chronic pelvic pain syndrome, reduces estrogen plasmatic levels and their concentration in the prostrate. Antifungal activity against *Candida* infections.		[65], [66]
Suloctidil	Used as a peripheral vasodilator, formerly in treating peripheral and cerebral vascular disorders; causes hepatotoxicity.		[67]

**Table 2:** Minimal inhibitory concentrations (MIC) *in vitro* and effective concentration (EC_50_) *in vivo* of compounds identified in the *C.elegans*-*C. albicans* screen.

Compounds	MIC *in vitro* (µg/mL)	MIC in literature for *C. albicans*	EC_50_ *in vivo* (µg/mL)
Amphotericin B	1.0	0.25–2.0 [41], [68]	2.0
Nystatin	4.5		17.8
Ketoconazole	4.4	0.12–16 [68]	8.9
Terconazole	0.06		0.11
Bifonazole	19.0	3.60* [50]	26.0
Butoconazole nitrate	0.6	8.0* [53]	1.1
Oxiconazole nitrate	4.7		9.4
Triadimefon	6.5		9.2
Dequalinium chloride	4.4		7.1
Valinomycin	12.2	0.49–62.5* [60]	24.4
Phenylmercuric acetate	7.4		14.8
Malachite green carbinol base	3.8		7.6
Ascomycin	14.6		29.3
FK-506 (Tacrolimus)	72.0	>3.12 [34]	72.0
Cyclosporin A	53.0	>12.5 [34]	105.8
Concanamycin A	1.7	>100* [40]	2.1
Mepartricin	12.6	0.18* [66]	25.1
Suloctidil	11.7		17.4

* The MIC was determined using a procedure other than the standard CLSI protocol.

Of the ten antimycotic compounds within the chemical library, all but three (flutrimazole, thiabendazole, and griseofulvin) were identified in the preliminary screen. All three of these compounds are used in topical treatment of fungal infections, and may have limited solubility in water, complicating their identification using the *C. elegans*-*C. albicans* “curing” assay. Furthermore, griseofulvin has no antifungal activity against *Candida* spp., as the fungus does not uptake the compound into the fungal cell, and therefore it would not be expected to be identified [22]. The compound thiabendazole is used agriculturally as a fungicide and has been used clinically in treatment of *Aspergillus flavus* [23], [24], however it is also nematocidial [25]. Several other antinematode compounds were represented in the chemical library including the clinically relevant agent albendazole and the avermectin, abamectin. These compounds exemplify three scenarios where a potential antifungal agent would be overlooked in the *C. elegans*-*C. albicans* antifungal discovery assay; 1) if the compound has limited solubility in water, 2) if the assayed compound has antifungal activity, as well as nematicidal activity, and 3) if the compound has activity against most fungi, although the fungus used in the screen (*C. albicans*) maybe unaffected by the compound.

**Figure 4:**
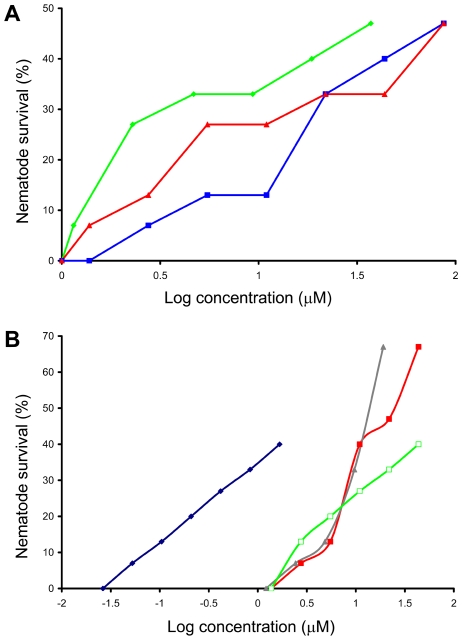
Survival of *C. albicans*-infected nematodes in the presence of seven compounds identified in the *C. elegans*–*C. albicans* “curing” assay. (A) Nematode survival in the presence of the three immunosuppressive compounds: ascomycin (green), cyclosporine A (red), and FK-506 (blue); (B) Nematode survival in the presence of four other compounds identified in the screen which demonstrated antifungal activity: concanamycin A (blue), dequalinium chloride (gray), triadimefon (red), and phenylmercuric acetate (green).

A number of known immuno-modulating agents were found to have antifungal activity in the *C. elegans* killing assay. This group includes FK-506, ascomycin, and cyclosporin A, all of which have previously been reported to have antifungal activity not only for *C. albicans*, but also other fungal pathogens such as *Cryptococcus neoformans*, *Aspergillus fumigatus*, and members of the zygomycetes [26]–[32]. These immunosupressives demonstrated weak antifungal activity when compared to clinically relevant antifungal agents, however they were able to confer ∼50% nematode survival at the highest concentration tested (Figure 4A). The target of these therapeutic drugs, calcineurin, is essential for virulence of *C. albicans* and is involved in a diverse array of other pathogenicity factors, including the ability to survive in serum and resistance to antifungal azole compounds [27]–[29]. The antifungal activity of these immunosuppressives, coupled with their ability to impede azole resistance, create (at least *in vitro*) a synergistic effect when used with clinically relevant antifungal agents [33], [34]. This synergism has been observed for many fungal pathogens, including azole resistant *C. albicans* isolates [31]–[33], [35]. Importantly, immunotherapy with calcineurin inhibitors does not select for *C. albicans* strains which are resistant to these compounds [36].

Among the hits was the potential promising compound dequalinium chloride which is a potent anti-tumor and protein kinase C (PKC) inhibitor (Figure 4B). In *Saccharomyces cerevisiae*, the Rho 1-PKC pathway (the cell integrity pathway) functions in cell wall biosynthesis and mating [5], [37], [38]; cell wall integrity is involved in *C. elegans* killing by fungi [5]. Another compound, triadimefon, also conferred a similar degree of nematode survival (Figure 4B). The fungicide triadimefon has no affect on DNA, RNA, or protein synthesis, and instead derives it antifungal activity by inhibiting ergosterol biosynthesis [39]. The potent H^+^ V- ATPase inhibitor concanamycin A was also identified, although it had been previously described as having antifungal properties [40]. This compound demonstrated the highest antifungal activity outside of the clinically used antifungal agents in the screen (Table 2 and Figure 4B). However, concanamycin A is highly toxic, limiting its use clinically. Mice injected with 1 mg per kilogram body mass interperitoneally die [40], however no toxicity was observed at the highest concentration tested (1.4 µg/mL).

### Determination of MIC and EC_50_ of compounds

Dose response curves were determined to calculate the minimal inhibitory concentration (MIC) and the effective dose that resulted in 50% survival of the nematodes (EC_50_) for 18 compounds identified in the screen that prevented growth of *C. albicans in vitro* (Table 2). The EC_50_ for most of the compounds was usually twice the MIC concentration, with a few exceptions. In this assay the MIC values may fluctuate from previously published reports, possibly due to differences in the liquid media used for each of the assays and in particular the time at which the cultures are monitored (two days in the established Clinical and Laboratory Standards Institute (CLSI) protocol for MIC determination versus five days in this assay). However, the standard antifungal used for optimization of the assay amphothericin B had an MIC value of 1.0 µg/mL, which is within the published range of 0.5–2.0 µg/mL [41].

Previously, three compounds were identified which prolonged nematode survival when infected with *C. albicans* [6]. Two of these compounds, caffeic acid phenethyl ester (CAPE) and enoxacin, also displayed antifungal activity when carried over into a murine model. However, of these two compounds only CAPE was able to inhibit *C. albicans* growth *in vitro*. The MIC of CAPE was 64 µg/mL, well above the 4 µg/mL concentration which was required to observe an increase in *C. elegans* survival [6], suggesting the compound maybe involved in a role besides inhibition of fungal growth, and thus conferring the observed increase in *C. elegans* survival. A similar trend was observed in a *C. elegans*-*Enterococcus faecalis* antibacterial drug discovery screen, where six classes of compounds displayed effective concentrations lower than the MIC [18]. Therefore, comparison to the MIC and the EC_50_ in the *C. elegans* assay may be used to identify compounds that have higher efficacy during infection than *in vitro*, as such compounds may affect virulence factors or modulate host immunity. Compounds that may fall into this class are often not identified in a traditional antifungal compound screen as the screen may rely solely on inhibiting microbial growth. Although the concentration of the compounds inside the nematode is probably lower than in the media, the ratio between the MIC and the EC_50_ provides a way to identify compounds that most likely have a higher efficacy *in vivo* than *in vitro*.

### Evaluation of compound toxicity

**Figure 5:**
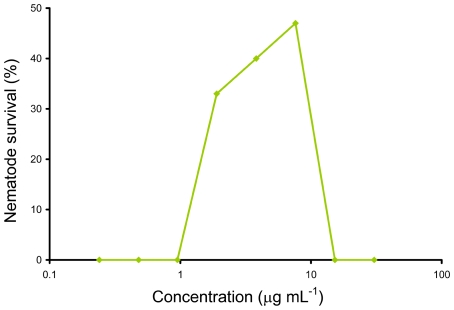
Dose-response of infected worms to malachite green carbinol base indicating toxicity beyond the EC_50_.

This screen also allowed simultaneous assessment of the compound's toxicity, as a decrease in nematode survival at higher compound concentrations despite no evidence of fungal growth *in vitro* or filamentation *in vivo* was an indication that the compound was toxic to the worm. In addition to the MIC and EC_50_ values, the dose-response experiments also detected toxic compounds such as malachite green carbinol base which is the carbinol form of malachite green, usually metabolized to the toxic leucomalachite green form [42]. In the initial portion of the curve, there was no worm survival up to a concentration of 0.95 µg/ml, and there were visible *C. albicans* filaments on dead worms. At concentrations greater than 0.95 µg/mL up to the EC_50_ concentration (7.62 µg/mL), worm survival increased, however at concentrations higher than the EC_50_ there was no survival of any worms (Figure 5). This was probably due to the toxic effect of the compound since *in vitro* growth of *C. albicans* in the wells was absent, and *in vivo* activity of the compound within the worms did not permit formation of *C. albicans* filaments penetrating from the cuticle into the surrounding medium.

### Concluding remarks

A high-throughput whole animal assay for the identification of compounds with antifungal efficacy has been developed using *C. elegans* as a heterologous host. The major developments include the use of the worm-dispensing instrument, the use of a standardized fungal inoculum, and the advance of using a co-inoculation procedure of *Candida* and *C. elegans* to the wells, thereby eliminating the labor-intensive infection. This facile *in vivo* model can now be used in high-throughput assays to evaluate large libraries of chemical compounds and avoid some of the main obstacles in current antifungal discovery, including host toxicity screening, costs, and time.

The overall excess cost attributable to candidemia is estimated at $1 billion per year [43], while the average cost of treating a single episode of candidemia is $34,123–44,536 (1997 U.S. dollars) [44]. Further complicating issues in treatment of candidemia is the emergence of drug resistant isolates. While most attention has been focused on antibacterial drug resistance, a similar phenomenon has occurred with fungi due to long term use of antifungal agents, particularly in patients who require these medications for the duration of their lives (eg. HIV positive patients, organ transplant recipients). Drug resistant *C. albicans* isolates have been isolated from patients which are resistant to the most recent class of antifungal compounds developed, the echinocandins [45], further highlighting the need for antifungal drug development [46]. The discovery of new antifungal agents may be a daunting but certainly crucial task.
