# Correction: Identification of Antifungal Compounds Active against *Candida albicans* Using an Improved High-Throughput *Caenorhabditis elegans* Assay

**DOI:** 10.1371/annotation/76699885-a47e-426c-accd-35576696c4f2

**Published:** 2010-04-06

**Authors:** Ikechukwu Okoli, Jeffrey J. Coleman, Emmanouil Tampakakis, W. Frank An, Edward Holson, Florence Wagner, Annie L. Conery, Jonah Larkins-Ford, Gang Wu, Andy Stern, Frederick M. Ausubel, Eleftherios Mylonakis

The third author's name was spelled incorrectly. The correct name is Emmanouil Tampakakis. The correct citation is: Okoli I, Coleman JJ, Tampakakis E, An WF, Holson E, et al. (2009) Identification of Antifungal Compounds Active against Candida albicans Using an Improved High-Throughput Caenorhabditis elegans Assay. PLoS ONE 4(9): e7025.doi:10.1371/journal.pone.0007025

